# Two novel *Planococcus* species isolated from *baijiu* pit mud with potential application in brewing

**DOI:** 10.3389/fmicb.2023.1139810

**Published:** 2023-05-11

**Authors:** Shuyue Hao, Qing Ren, Jiaxuan Wang, Liya Li, Mingquan Huang

**Affiliations:** Key Laboratory of Brewing Molecular Engineering of China Light Industry, Beijing Technology and Business University, Beijing, China

**Keywords:** *Planococcus beigongshangi*, *Planococcus beijingensis*, *baijiu*, *huangjiu*, application

## Abstract

Two novel Gram-positive bacteria, designated strains REN8^T^ and REN14^T^, were isolated from *baijiu* pit mud in Sichuan Province, China. REN8^T^ achieved the best growth at 37°C, a pH of 8.0, and a NaCl concentration of 2%, while REN14^T^ displayed optimal growth at 37°C, a pH of 6.0, and a NaCl concentration of 1%. 16S rRNA and genomic phylogenetic analysis showed that REN8^T^ and REN14^T^ were clustered with the genus *Planococcus*. The genomic DNA G + C contents of REN8^T^ and REN14^T^ were 46.7 and 45.1 mol%, respectively. The dDDH and ANI values were 24.5 and 80.43% between REN8^T^ and *P. salinarum* (the most closely related type strain) and 25.1 and 82.42% between REN14^T^ and *P. soli* (the most closely related type strain). Genomic analysis showed that several carbohydrate-active enzymes and secondary metabolite gene clusters existed in REN8^T^ and REN14^T^. Chemotaxonomic characteristics of REN8^T^ and REN14^T^ included major fatty acids, predominant menaquinones, and polar lipids, all of which were consistent with the genus *Planococcus*. Based on the polyphasic taxonomic method, these two strains represent two novel species of the genus *Planococcus*; the name *Planococcus beigongshangi* sp. nov. is proposed for the type strain REN8^T^ (=JCM 33964^T^ = GDMCC 1.2213^T^), and the name *Planococcus beijingensis* sp. nov. is proposed for the type strain REN14^T^ (=JCM 34410^T^ = GDMCC 1.2209^T^). The addition of REN8^T^ and REN14^T^ might improve the quality of *huangjiu* by considerably increasing the amino acid nitrogen content and acidity and decreasing the bioamine content, with no significant change in alcohol content.

## Introduction

The genus *Planococcus* was first described by Migula in 1894 (Approved Lists 1980) (Kocur et al., 1970). To date, the genus *Planococcus* encompasses 24 species with validly published and correct names [LPSN—List of Prokaryotic names with Standing in Nomenclature (dsmz.de)]. The genus *Planococcus* is widely distributed in different regions and environments, especially marine and high salt areas. All members of the genus are characterized as aerobic, shaped as cocci or short rods, and generally motile, Gram-positive to Gram-variable and nonendospore forming (Gupta and Patel, 2020).

*Baijiu* is a traditional fermented alcoholic beverage in China (Xu et al., 2021). Pit mud, as a place for anaerobic fermentation of *baijiu*, contains abundant microbial resources, which play crucial roles in the unique flavor formation of *baijiu* (Qian et al., 2021). Pit mud can provide a suitable environment for the survival of microorganisms, and the microbial community in pit mud undergoes complex energy metabolism, which is very important to the quality of *baijiu* (Wang et al., 2020). Pit mud can be continuously used for decades; therefore, recycled pit mud forms a unique and complex microbial community (Tao et al., 2017). Screening and identification of new microbial resources from pit mud are helpful for further understanding the unique flavor of *baijiu*. Moreover, numerous microbial resources have additional functions in other fields, such as medicine and biocontrol (Yu et al., 2022). *Huangjiu* is a kind of brewing wine made with wheat and water as raw materials through the joint metabolism, fermentation, and interaction of molds, yeast, and bacteria through a unique process (Yu et al., 2023). Different from *baijiu*, *huangjiu* belongs to fermented wine and *baijiu* belongs to distilled wine. Therefore, bacteria were isolated and cultured from pit mud to uncover new bacterial species and study the relationship between bacteria and *huangjiu* flavor components.

In this study, two new members of the genus *Planococcus* were isolated from *baijiu* pit mud (Supplementary Figure S1 shows the isolation site of the two strains and *baijiu* production process) in Sichuan Province, China. The two new strains named REN8^T^ and REN14^T^ were identified based on a polyphasic taxonomic approach. Finally, the names *Planococcus beigongshangi* for strain REN8^T^ and *Planococcus beijingensis* for strain REN14^T^ were proposed.

## Materials and methods

### Isolation and culture conditions

Samples of *baijiu* pit mud were collected in 2019 from Sichuan Province, China (30°05′N 102°54′E). Samples were treated and diluted as previously described (Sun et al., 2021). Then, the mud suspension was spread on trypticase soy agar (TSA) and R2A agar medium. A light yellow single colony named strain REN8^T^ was isolated after incubating on TSA at 30°C for 2 days. Another light yellow single colony named strain REN14^T^ was isolated after incubating on R2A at 37°C for 2 days. REN8^T^ and REN14^T^ were preserved in a 30% glycerol suspension at −80°C for further analysis.

### 16S rRNA gene amplification and phylogenetic analysis

Genomic DNA of REN8^T^ and REN14^T^ was extracted by a TIANGEN Bacterial DNA Kit (TIANGEN Biotech Beijing Co., Ltd., Beijing, China). Universal primer pairs of 27F and 1492R were used to amplify the 16S rRNA gene of REN8^T^ and REN14^T^ by PCR with the following program: 94°C for 5 min; 35 cycles of 94°C for 30 s, 55°C for 30 s, and 72°C for 3 min; and a final extension at 72°C for 10 min. After electrophoresis detection, the PCR products were sequenced by Sangon Biotech Co., Ltd. (Shanghai, China). The NCBI BLAST algorithm and EzBioCloud server were used to analyze the 16S rRNA gene similarity of REN8^T^ and REN14^T^ (Yoon et al., 2017). 16S rRNA gene sequences of REN8^T^, REN14^T^ and other closely related type strains were aligned with the CLUSTALX program (Thompson et al., 1997). Phylogenetic analysis of REN8^T^ and REN14^T^ was conducted by the neighbor-joining method (Saitou and Nei, 1987) using the Kimura two-parameter model with 1,000 replicate bootstrap values by MEGA 11 software (Kimura, 1980; Tamura et al., 2021).

### Genomic sequencing and analysis

Whole-genome sequencing of REN8^T^ and REN14^T^ was conducted at Shanghai Majorbio Bio-Pharm Technology Co., Ltd. (Shanghai, China). Then, SOAPdenovo was used to assemble the clean reads (Li et al., 2008, 2010). The average nucleotide identity (ANI) between REN8^T^ and REN14^T^, REN8^T^ and the closest species *Planococcus salinarum* ISL-16^T^ (Yoon et al., 2010), and REN14^T^ and the closest species *Planococcus soli* XN13^T^ (Luo et al., 2014) were calculated using the ANI calculator (Yoon et al., 2017). The digital DNA–DNA hybridization (dDDH) values were also determined by Genome-to-Genome Distance Calculator 2.1 (Auch et al., 2010). The genomic DNA G + C content of REN8^T^ and REN14^T^ was calculated (Aziz et al., 2008). The genomic phylogenetic analysis among REN8^T^, REN14^T^ and related species was also constructed using PhyloPhlAn software (Asnicar et al., 2020).

Genes from REN8^T^ and REN14^T^ were annotated to the NR, Swiss-Prot, Pfam, COG, GO, and KEGG databases (Ashburner et al., 2000; Bairoch and Apweiler, 2000; Kanehisa and Goto, 2000; Deng et al., 2006; Jensen et al., 2008; Finn et al., 2014). The genomic characteristics of REN8^T^ and REN14^T^ were analyzed by CGView software (Stothard and Wishart, 2005). The numbers of carbohydrate-active enzymes (CAZy) and secondary metabolite gene clusters were analyzed by the CAZy database and antiSMASH, respectively (Lombard et al., 2014; Blin et al., 2021).

### Phenotypic characterization

Morphological characteristics of REN8^T^ and REN14^T^ were observed under light and transmission electron microscopes. An anaerobic system (Thermo Scientific, United States) filled with tri-mixture anaerobic gas (85:5:10 mixture of N_2_, CO_2_, and H_2_) was used to evaluate the anaerobic growth ability of REN8^T^ and REN14^T^. The Gram reaction of REN8^T^ and REN14^T^ was performed according to the nonstaining method (Buck, 1982). The morphology and color of REN8^T^ and REN14^T^ on TSA medium were observed by the naked eye. Four media, LB (Luria-Bertani) agar, R2A agar, NA (nutrient agar) and TSA, were used to assess the growth ability of REN8^T^ and REN14^T^. The growth ability of REN8^T^ and REN14^T^ at different temperatures (10, 20, 28, 30, 37, 42, 45, 50, and 55°C), different NaCl tolerances (0, 1, 2, 3, 4, 5, 6, 7, 8, 9, 10, 11, 15, and 20% w/v), and different pH values (3–12, with the pH increments using the following biological buffer system: pH 3.0–5.0, 2 M sodium acetate; pH 6.0–7.0, 1 M KH_2_PO_4_, 1 M NaOH; pH 8.0, 1 M Tris–HCl; pH 9.0–10.0, 1.5 M NaHCO_3_, 1.5 M Na_2_CO_3_; and 11–12, 0.5 M NaOH) (Xu et al., 2005) were determined. The catalase activity and hydrolysis capacity of REN8^T^ and REN14^T^ were investigated as previously described (Sun et al., 2012). API ZYM and API 20NE kits (bioMérieux, France) were used to investigate the enzyme activities and biochemical characteristics of REN8^T^ and REN14^T^. GENIII Micro Plates (Biolog) were used to detect the basic biochemical tests and carbon source oxidation tests of REN8^T^ and REN14^T^.

### Chemotaxonomic characterization

The fatty acids of REN8^T^ and REN14^T^ were treated and detected by the Microbial Identification System (MIDI) (Sasser, 1990). The polar lipids of REN8^T^ and REN14^T^ were investigated by two-dimensional thin-layer chromatography (TLC) (Minnikin et al., 1984). Respiratory quinones of REN8^T^ and REN14^T^ were extracted, examined and analyzed by high-performance liquid chromatography (HPLC) (Hu et al., 2001).

### Preparation of wheat *qu* and fortified *qu* for *huangjiu*

Wheat qu is produced by naturally cultivating or purebred breeding microorganisms on broken raw wheat grains. Wheat was ground, and 20–30% water was added. Then, the mixtures were evenly stirred, treaded and stacked. Wheat *qu* was finally obtained after natural fermentation at 45–50°C.

One kilogram of millet and 3 kg of water were mixed and thoroughly cooked. After adding 0.15% saccharifying enzyme at 60°C for 30 min, REN8^T^ and REN14^T^ were inoculated and cultured for 48 h at 37°C. The fortified *qu* was prepared as follows: wheat was ground, and 20–30% water, 1% REN8^T^ and REN14^T^ culture solutions were added. Then, the mixtures were evenly stirred, treaded and stacked, and natural fermentation was performed at 45–50°C. Finally, *qu* fortified with REN8^T^ was named QR8, *qu* fortified with REN14^T^ was named QR14, and wheat *qu* was named Qu1.

### *Huangjiu* brewing and measured indices

*Huangjiu* was brewed using the following steps: millet cooking, saccharification, fermentation, clarification and sterilization.

Several indices of *huangjiu*, including the bioamine content, volatile substances, amino acid nitrogen content, nonsugar solid content, total sugars, alcohol, and acidity, were measured. The bioamine content was detected by HPLC according to the national standard (GB 5009.208–2016). The volatile substances in *huangjiu* were determined as previously described by Xing and Ren (2019). Amino acid nitrogen, nonsugar solid content, total sugars, alcohol and acidity were detected according to national standards (GB/T 13662–2018). The differences in bioamine content, volatile substances, amino acid nitrogen, nonsugar solid content, total sugars, alcohol and acidity between *huangjiu* brewed by wheat *qu* and *huangjiu* brewed by fortified *qu* were compared. All experiments were repeated three times, and a significance analysis with one-way ANOVA in SPSS Statistics was also conducted.

## Results

### Phylogenetic analysis

**Figure 1:**
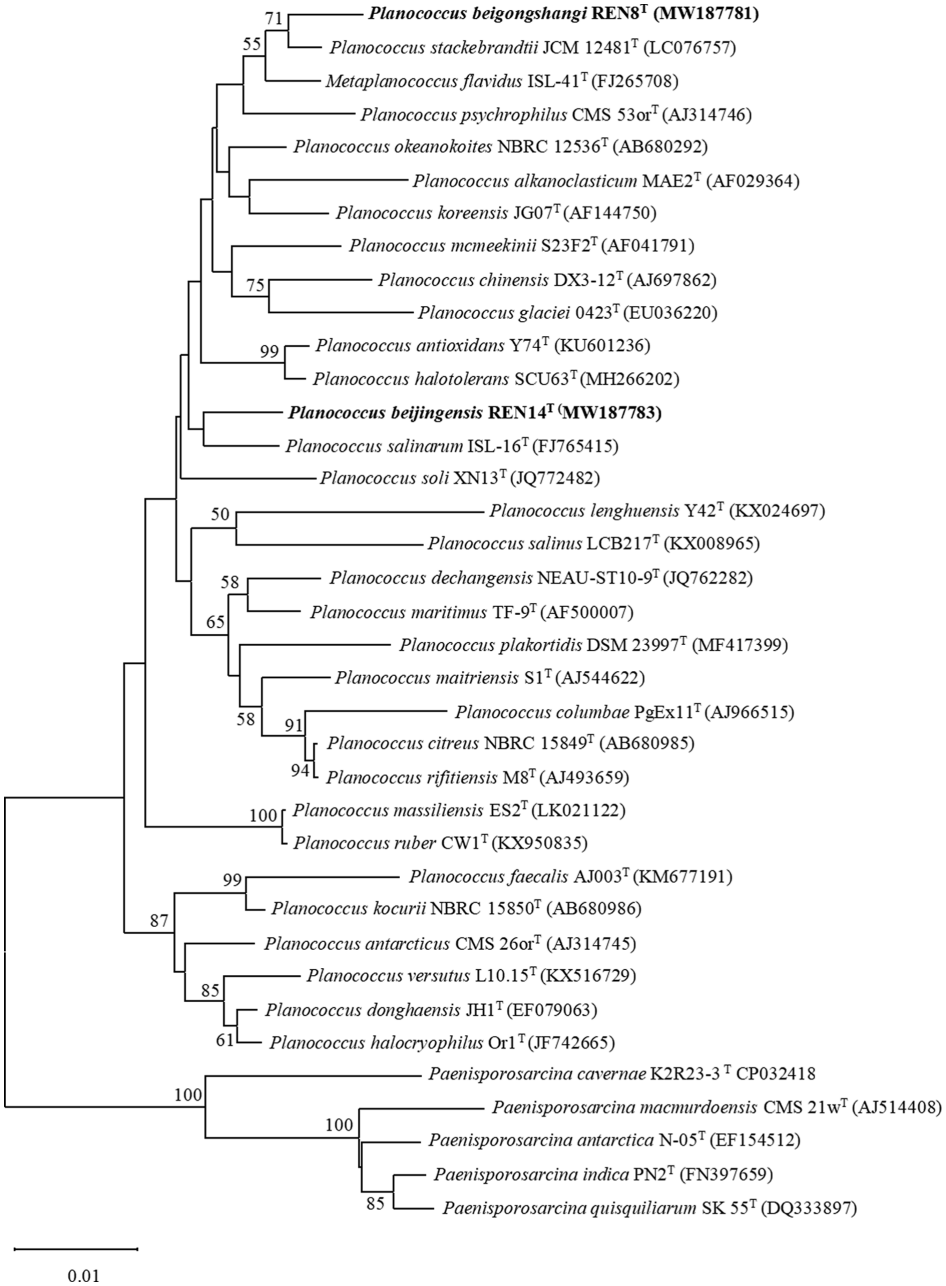
Phylogenetic analysis of the relationships of strains REN8^T^ and REN14^T^ and closely related species based on 16S rRNA gene sequences. The Kimura two-parameter model was used based on neighbor-joining analysis. Numbers at nodes are bootstrap values (%) based on 1,000 replications. Bar, 0.002 substitutions per nucleotide position.

**Figure 2:**
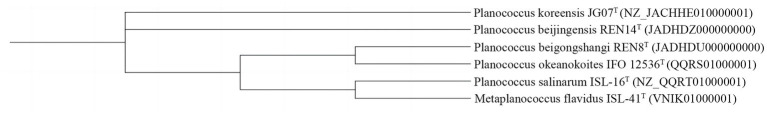
Phylogenetic analysis of the relationships of strains REN8^T^ and REN14^T^ and closely related species based on genomic analysis.

The 16S rRNA gene sequence of REN8^T^ was 1,514 bp. The 16S rRNA gene sequence of REN14^T^ was 1,407 bp. Based on the 16S rRNA phylogenetic analysis, REN8^T^ and REN14^T^ fell within the genus *Planococcus* (Figure 1). Genome-based phylogenetic analysis showed that REN8^T^ and REN14^T^ were clustered with the genus *Planococcus* (Figure 2).

### Genomic analysis

Genome information of REN8^T^ and REN14^T^ is shown in Table 1 (RAST Server—RAST Annotation Server).^1^ The genomic DNA G + C contents of REN8^T^ and REN14^T^ were 46.7 and 45.1 mol%, respectively, which fell within the genus *Planococcus* (34.8–51.0 mol%). The dDDH values between REN8^T^ and *P. salinarum* and between REN8^T^ and REN14^T^ were 24.5 and 20.1%, respectively. The dDDH value between REN14^T^ and *P. soli* was 25.1%. The ANI values between REN8^T^ and *P. salinarum* and between REN8^T^ and REN14^T^ were 80.43 and 74.7%, respectively. The ANI value between REN14^T^ and *P. soli* was 82.42%. The dDDH and ANI values of REN8^T^ and REN14^T^ and other strains of the same genus are shown in Table 2 [Type Strain Genome Server (dsmz.de)]. Based on the dDDH and ANI analysis, REN8^T^ and REN14^T^ were regarded as two novel species in the genus *Planococcus*.

**Table 1:** Genome features of REN8^T^ and REN14^T^.

Statistic	REN8^T^	REN14^T^
Sequence size	3,214,327	3,713,797
Number of contigs	32	21
GC content (%)	46.7	45.1
Shortest contig size	306	364
Median sequence size	6,749	65,329
Mean sequence size	100447.7	176847.5
Longest contig size	863,506	1,291,737
N50 value	576,022	465,862
L50 value	3	3

**Table 2:** Digital DNA–DNA hybridization (dDDH) and average nucleotide identity (ANI) values between REN8^T^and REN14^T^ and type strains of closely related species.

Reference strain	*Planococcus beigongshangi* REN8^T^			*Planococcus beijingensis* REN14^T^		
	dDDH (in %)	ANI (in %)	G + C content difference (in %)	dDDH (in %)	ANI (in %)	G + C content difference (in %)
*P. salinarum* DSM 23820^T^	24.5	80.43	1.3	19.8	78.21	2.84
*P. mcmeekinii* DSM 13963^T^	24.3	81.57	1.06	19.7	78.35	0.49
*P. halotolerans* SCU63^T^	23.3	80.79	2.1	19.7	78.26	0.56
*P. okeanokoites* IFO 12536^T^	23.1	80.90	1.13	20	78.30	0.42
*P. koreensis* DSM 15895	19.6	77.83	0.9	19.9	78.82	2.44
*P. chinensis* DSM 17276^T^	19.5	78.53	3.4	19.7	78.25	4.94
*P. glaciei* CGMCC 1.6846^T^	19.4	78.17	0.07	19.8	78.44	1.61
*P. massiliensis* ES2^T^	19.4	78.01	0.72	19.5	78.00	0.83
*P. soli* C23T^T^	19.3	78.43	1.88	25.1	82.48	0.33
*P. salinus* LCB217^T^	19.3	77.57	0.55	19.5	77.67	0.99
*P. versutus* L10.15^T^	19.2	78.01	7.28	21.3	79.69	5.74
*P. antarcticus* DSM 14505^T^	19.2	77.85	3.67	24.5	81.66	2.12
*P. kocurii* ATCC 43650^T^	19.1	77.86	5.76	22.6	80.62	4.21
*P. maitriensis* S1^T^	19.1	77.72	3.23	19	77.42	4.78
*P. donghaensis* DSM 22276^T^	19	77.59	6.59	21.7	80.24	5.04
*P. faecalis* CECT 8759^T^	19	77.64	5.84	22.7	80.57	4.3
*P. halocryophilus* DSM 24743^T^	18.7	77.71	6.78	21.8	80.10	5.24

**Figure 3:**
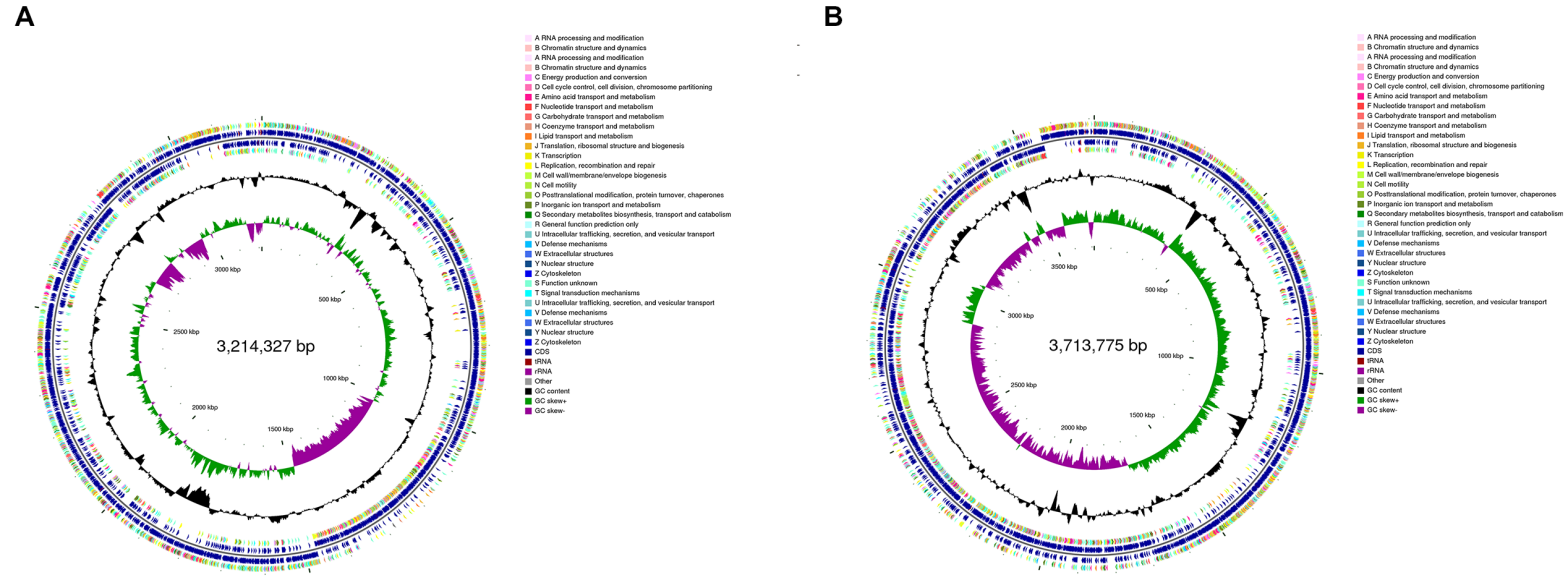
Circular plot from the genomes of REN8^T^ and REN14^T^. Different loop levels represent relevant genomic information. **(A)** Genome of REN8^T^. **(B)** Genome of REN14^T^.

The genomic information of REN8^T^ and REN14^T^ is shown in Figures 3A,B. In total, 3,130 (NR), 2,454 (Swiss-Prot), 2,685 (Pfam), 2,806 (COG), 618 (GO), and 1,676 (KEGG) genes were annotated in REN8^T^. Twenty-three carbohydrate esterases, 23 glycosyl transferases, and 14 glycoside hydrolases were found in REN8^T^. Two terpenes and one T3PKS secondary metabolite cluster were found in REN8^T^. A total of 3,583 (NR), 2,922 (Swiss-Prot), 3,154 (Pfam), 3,275 (COG), 2,656 (GO), and 1,946 (KEGG) genes were annotated in REN14^T^. There were 28 glycosyl transferases, 26 carbohydrate esterases, and 25 glycoside hydrolases in REN14^T^. REN14^T^ had one cluster of lanthipeptide-class-II secondary metabolites and two terpenes.

### Phenotypic characteristics

The basic characteristics of REN8^T^ were as follows: Gram-positive, aerobic, and motile bacteria with short rods. The size of REN8^T^ ranged from 0.4 to 0.6 m in width and 0.8 to 1.3 m in length (Supplementary Figure S2A). The diameter of REN14^T^ was 1.0–2.0 μm (Supplementary Figure S2B), with the basic characteristics of Gram-positive, coccoid, aerobic and motile bacteria. After incubation for 2 days on TSA medium, colonies of REN8^T^ and REN14^T^ were circular, slightly convex, glistening, smooth and light yellow in color. REN8^T^ and REN14^T^ were able to grow on LB agar, R2A agar, TSA medium, and NA medium, and TSA medium was optimal. The growth temperature, pH and NaCl concentration range of REN8^T^ was 28–37°C, 7.0–11.0 and 0–6% (w/v), respectively, with the optimal conditions being 37°C, a pH of 8.0 and 2% NaCl. Meanwhile, REN14^T^ exhibited a growth range of 20–45°C, a pH of 5.0–12.0 and 0–15% (w/v) NaCl, with the optimal conditions being 37°C, a pH of 6.0 and 1% NaCl. The biochemical characteristics of REN8^T^, REN14^T^ and other highly related species are shown in Table 3.

**Table 3:** Comparison of the phenotypic characteristics of strains REN8^T^ and REN14^T^ and type strains of closely related species.

Characteristic	1	2	3	3	4	5	6	7	8
Growth temperature (°C)	28–37	20–45	4–38	4–37	20–37	12–43	4–39	0–30	15–37
NaCl tolerance (%, w/v)	0–6	0–15	0–13	0.5–13	0–7	0–10	0–7	0–12	0–7
Utilization									
α-D-glucose	−	+	−	−	−	+	W	−	−
α-D-lactose	−	+	−	−	−	−	+	−	−
D-cellobiose	−	−	−	−	−	−	+	−	−
D-mannitol	−	+	−	−	−	−	−	−	−
D-melibiose	−	−	−	−	−	−	+	−	−
Gelatin	−	W	−	−	+	+	+	+	−
Starch	+	+	−	−	−	−	−	−	−
Sucrose	−	+	−	−	−	−	−	−	+
Enzyme activity									
D-mannose	+	−	−	−	−	−	−	−	−
L-arabinose	+	−	−	−	−	−	−	−	+
Oxidase	+	+	+	+	W	−	−	+	+
Potassium nitrate	−	−	−	−	−	+	−	−	−
Predominant menaquinones	MK-8,7	MK-7, 8, 6	MK-8,7	MK-8,7	MK-8,7	MK-8,7	MK-8, 7, 6	MK-8,7	MK-7, 8, 6
G + C content (mol%)	46.7	45.1	48.3	45.9	46.3	34.8	47.0	44.5	51.0

Strains: 1, REN8^T^; 2, REN14^T^; 3, *Planococcus salinarum* ISL-16^T^ (Yoon et al., 2010); 4, *Planococcus okeanokoites* IFO 12536^T^ (Nakagawa et al., 1996); 5, *Planococcus chinense* DX3-12^T^ (Dai et al., 2005; Gupta and Patel, 2020); 6, *Planococcus koreensis* JG07^T^ (Yoon et al., 2001; Gupta and Patel, 2020); 7, *Planococcus psychrophilum* CMS 53or^T^ (Reddy et al., 2002); 8. *Planococcus plakortidis* AS/ASP6 (II)^T^ (Kaur et al., 2012).

+, Positive; −, negative; W, weakly positive.

### Chemotaxonomic characteristics

Results similar to those of other members of the genus *Planococcus* were detected in REN8^T^ and REN14^T^. The major fatty acids of REN8^T^ were iso-C_15:0_ (33.15%), antesio-C_15:0_ (23.82%), iso-C_17:0_ (13.36%), and antesio-C_17:0_ (9.97%), and those of REN14^T^ were iso-C_15:0_ (45.62%), antesio-C_15:0_ (26.56%), iso-C_17:0_ (9.38%), and antesio-C_17:0_ (6.47%). Although the fatty acid compositions of REN8^T^ and REN14^T^ were similar to those of the reference strains, their proportions varied (Table 4). The cellular polar lipids of REN14^T^ were DPG, PE and PG, and REN8^T^ had polar lipids of DPG, PE, PG and AL (Supplementary Figure S3). The predominant menaquinones of REN8^T^ were MK-8:MK-7 = 28:72, with REN14^T^ having menaquinones of MK-8:MK-7:MK-6 = 44:45:11.

**Table 4:** Comparison of the fatty acid (%) profiles of strains REN8^T^ and REN14^T^ and type strains of closely related species.

Fatty acid	1	2	3	4	5	6	7	8
C_16:0_	4.28	2.84	–	4.7	1.5	23.8	4.4	1.9
Antesio-C_15:0_	23.82	26.56	44.8	14.0	49.7	32.9	41.3	34.2
Antesio-C_17:0_	9.97	6.47	10.8	–	2.8	3.7	7.3	5.5
Iso-C_14:0_	1.20	1.9	4.2	33.9	10.4	7.5	3.2	9.6
Iso-C_15:0_	33.15	45.62	2.7	2.9	3.8	5.2	5.6	13.2
Iso-C_16:0_	5.80	3.88	7.5	28.1	4.1	8.1	8.1	11.7
Iso-C_17:0_	13.36	9.38	2.5	–	–	2.7	–	2.9

Strains: 1, REN8^T^; 2, REN14^T^; 3, *Planococcus salinarum* ISL-16^T^ (Yoon et al., 2010); 4, *Planomicrobium okeanokoites* IFO 12536^T^ (Nakagawa et al., 1996); 5, *Planomicrobium chinense* DX3-12^T^ (Dai et al., 2005); 6, *Planococcus koreensis* JG07^T^ (Yoon et al., 2001; Gupta and Patel, 2020); 7, *Planococcus psychrophilum* CMS 53or^T^ (Reddy et al., 2002); 8, *Planococcus plakortidis* AS/ASP6 (II)^T^ (Kaur et al., 2012).

–, not available.

### The effect of fortified *qu* on *huangjiu* indices

Compared with wheat *qu*, fortified *qu* significantly reduced the content of bioamines. QR8 and QR14 reduced bioamines by 18.7 and 20.7%, respectively. Among these bioamines, histamine, spermine and spermidine were markedly reduced (Table 5).

**Table 5:** Comparison of the bioamine content of wheat *qu* (Qu1) and wheat *qu* inoculated with REN8^T^ (QR8) and REN14^T^ (QR14).

Bioamine	Qu1	QR8	QR14
Putrescine	3.95 ± 0.12a	3.88 ± 0.09a	4.01 ± 0.11a
Cadaverine	0.43 ± 0.01a	0.37 ± 0.02a	0.35 ± 0.08a
Histamine	10.48 ± 1.05a	8.33 ± 0.68b	8.02 ± 0.77b
Tyramine	0.56 ± 0.02a	0.60 ± 0.01a	0.61 ± 0.01a
Spermine	7.56 ± 0.18a	5.12 ± 0.14b	5.35 ± 0.17b
Spermidine	8.38 ± 0.66a	6.18 ± 0.42b	6.01 ± 0.53b
Phenylethylamine	4.92 ± 0.07a	5.03 ± 0.12a	4.45 ± 0.16a
Total content	36.28 ± 1.59a	29.51 ± 2.02b	28.76 ± 1.94b

Qu1, wheat *qu*; QR8, *qu* fortified with REN8^T^; QR14, *qu* fortified with REN14^T^.

For volatile substances, 104 volatile substances were detected in Qu1, while 106 and 109 kinds of volatile substances were detected in QR8 and QR14, respectively. Except for acid substances, other volatile substances and total content were not significantly altered among different types of *huangjiu*, which indicated that the addition of REN8^T^ and REN14^T^ did not influence the flavor quality of *huangjiu* fermentation (Tables 6, 7).

**Table 6:** Comparison of the varieties of flavor components in wheat *qu* (Qu1) and wheat *qu* inoculated with REN8^T^ (QR8) and REN14^T^ (QR14).

Flavor component	Qu1	QR8	QR14
Alkane	18	21	19
Alcohol	32	33	33
Ester	26	27	26
Ketone	4	3	5
Aldehyde	4	4	4
Acid	5	5	6
Pyrazine	1	2	2
Phenol	4	3	3
Others	10	8	11
Total	104	106	109

Qu1, wheat *qu*; QR8, *qu* fortified with REN8^T^; QR14, *qu* fortified with REN14^T^.

**Table 7:** Comparison of flavor component content of wheat *qu* (Qu1) and wheat *qu* inoculated with REN8^T^ (QR8) and REN14^T^ (QR14).

Flavor component	Qu1	QR8	QR14
Alkane	89.37 ± 4.85a	92.16 ± 5.24a	94.44 ± 6.01a
Alcohol	147.23 ± 4.09a	151.71 ± 5.26a	151.71 ± 5.26a
Ester	49.73 ± 3.62a	51.42 ± 3.66a	53.12 ± 3.74a
Ketone	0.68 ± 0.03a	0.71 ± 0.013a	0.65 ± 0.01a
Aldehyde	0.20 ± 0.01a	0.22 ± 0.03a	0.21 ± 0.01a
Acid	2.19 ± 0.01a	3.12 ± 0.02b	3.10 ± 0.01b
Pyrazine	0.02 ± 0.001a	0.03 ± 0.002a	0.03 ± 0.001a
Phenol	7.47 ± 0.38a	8.01 ± 0.22a	7.58 ± 0.33a
Others	8.52 ± 0.81a	6.69 ± 0.75a	7.03 ± 0.82a
Total	302.41 ± 8.52a	314.06 ± 6.79a	317.87 ± 7.06a

Qu1, wheat *qu*; QR8, *qu* fortified with REN8^T^; QR14, *qu* fortified with REN14^T^.

For other indices, the contents of alcohol, nonsugar solids and total sugars were not significantly altered after using QR8 and QR14, while amino acid nitrogen and acidity were dramatically improved (Table 8).

**Table 8:** Quality comparison of wheat *qu* (Qu1) and wheat *qu* inoculated with REN8^T^ (QR8) and REN14^T^ (QR14).

Indexes	Qu1	QR8	QR14
Total acid/g•L^−1^	4.89 ± 0.72a	6.23 ± 0.46b	6.58 ± 0.55b
Total sugar/g•L^−1^	71.36 ± 3.61a	75.29 ± 3.05a	74.57 ± 3.67a
Non-sugar solid content /g•L^−1^	18.33 ± 1.73a	17.84 ± 2.06a	18.03 ± 1.89a
Alcohol content/%	10.65 ± 2.41a	11.44 ± 1.54a	11.36 ± 1.78a
Amino acid nitrogen/g•L^−1^	0.31 ± 0.01a	0.49 ± 0.02b	0.51 ± 0.01b

Qu1, wheat *qu*; QR8, *qu* fortified with REN8^T^; QR14, *qu* fortified with REN14^T^.

## Discussion

In this study, two novel species were isolated and identified. Based on the polyphasic taxonomic method described above, including phylogenetic analysis, genomic analysis, phenotypic characteristics and chemotaxonomic characteristics, strains REN8^T^ and REN14^T^ are two novel species within the genus *Planococcus*. Therefore, two novel species named *Planococcus beigongshangi* sp. nov. and *Planococcus beijingensis* sp. nov. are proposed.

Excessive bioamine consumption is harmful to health. In *huangjiu* brewing, controlling the content of bioamines is very important. Screening of specific strains that could effectively decompose biogenic amines is crucial to controlling the content of bioamines in *huangjiu* brewing. In this research, the addition of REN8^T^ and REN14^T^ could improve the quality of *huangjiu*, significantly increasing amino acid nitrogen content and acidity and decreasing the bioamines content, with flavoring substances and the alcohol content showing no obvious alteration.

In terms of reducing bioamines, the addition of REN8^T^ and REN14^T^ could significantly reduce the content of bioamines. This could be because REN8^T^ and REN14^T^ effectively inhibited the growth of some microorganisms that produce bioamines, resulting in a decrease in bioamine content in *huangjiu*. The addition of REN8^T^ and REN14^T^ may influence the microecology of the brewing process, causing some infectious microorganisms to be inhibited and the content of amino acid nitrogen and acidity to be improved.

## Conclusion

Two novel Gram-positive bacteria, designated strains REN8^T^ and REN14^T^, were isolated from *baijiu* pit mud in Sichuan Province, China. 16S rRNA and genomic phylogenomic analysis showed that REN8^T^ and REN14^T^ belonged to the genus *Planococcus*. The dDDH values between REN8^T^ and *P. salinarum* and between REN14^T^ and *P. soli* were 24.5 and 25.1%, respectively. The ANI values between REN8^T^ and *P. salinarum* and between REN14^T^ and *P. soli* were 80.43 and 82.42%, respectively. Combined with morphological, physiological, biochemical properties, and chemotaxonomic characteristics including major fatty acids, polar lipids and predominant menaquinones, strain REN8^T^ represents a novel species, and the name *Planococcus beigongshangi* sp. nov. was proposed for the type strain REN8^T^ (=JCM 33964^T^ = GDMCC 1.2213^T^) (Table 9). Strain REN14^T^ also represents a novel species, and the name *Planococcus beijingensis* sp. nov. is proposed for the type strain REN14^T^ (=JCM 34410^T^ = GDMCC 1.2209^T^) (Table 10). The addition of REN8^T^ and REN14^T^ to *huangjiu* could improve its quality by increasing the amino acid nitrogen content and acidity while decreasing the bioamine content, and the flavoring substances and alcohol content were not obviously altered.

**Table 9:** Description of *Planococcus beigongshangi* sp. nov.

Species name	*Planococcus beigongshangi*
Genus name	*Planococcus*
Specific epithet	*beigongshangi*
Species status	sp. nov.
Species etymology	bei.gong.shang’i. N.L. gen. n. beigongshangi of Beigongshang, Chinese abbreviated name of Beijing Technology and Business University
Description of the new taxon and diagnostic traits	Cells are Gram-staining-positive, short rods, aerobic and motile (0.4–0.6 μm in width and 0.8–1.3 μm in length). Colonies on TSA are circular, slightly convex, glistening, smooth and light yellow in color after incubation for 2 days at 30°C. Cells are catalase positive and can hydrolyze starch and milk but not peptonisation. Growth occurs between 28 and 37°C (optimal growth at 37°C) and at pH 7.0–11.0 (optimal growth at pH 8.0). Growth occurs with 0–6% NaCl (optimal growth with 2% NaCl).
Positive tests with Biolog	Utilize dextrin, Acetoacetic acid, Acetic acid. Weakly utilize D-mannose, L-galactonic acid lactone, D-glucuronic acid, Tetrazolium violet, Tetrazolium blue, Tween 40, Propionic acid
Negative tests with Biolog	D-cellobiose, D-trehalose, Gentiobiose, Sucrose, D-turanose, Stachyose, D-raffinose, α-D-lactose, D-melibiose, β-methyl-D-glucoside, D-salicin, N-acetyl-D-glucosamine, N-Acetyl-β-Dmannosamine, N-acetyl-D-galactosamine, N-acetyl neuraminic acid, α-D-glucose, D-fructose, D-galactose, 3-methyl glucose, D-fucose, L-fucose, L-rhamnose, Inosine, 1% sodium lactate, Fusidic acid, D-serine, D-sorbitol, D-mannitol, D-arabitol, myo-inositol, Glycerol, D-glucose-6-PO4, D-fructose-6-PO4, D-aspartic acid, Troleandomycin, Rifamycin SV, Minocycline, Gelatin, Glycyl-L-prolin, L-alanine, L-arginine, L-aspartic acid, L-glutamic acid, L-histidine, L-pyroglutamic acid, L-serine, Lincomycin, Guanidine HCl, Niaproof 4, Pectin, D-galacturonic acid, D-gluconic acid, Glucuronamide, Mucic acid, Quinic acid, D-saccharic acid, Vancomycin, p-Hydroxy-Phenylacetic acid, Methyl pyruvate, D-lactic acid methyl ester, L-lactic acid, Citric acid, α-keto-glutaric acid, D-malic acid, L-malic acid, Bromo-succinic acid, Nalidixic acid, Lithium chloride, Potassium tellurite, γ-amino-butryric acid, α-hydroxy-butyric acid, β-hydroxy-D, Lbutyric acid, α-keto-butyric acid, Formic acid, Aztreonam, Sodium butyrate, Sodium bromate
Positive tests with API	Alkaline phosphatase, Esterase (C4), Esterase lipase (C8), Lipase (C14), Leucine arylamidase, Valine arylamidase, Cystine arylamidase, Trypsin, α-chymotrypsin, Cystine arylamidase, Trypsin, α-chymotrypsin, naphthol-AS-BI-phosphohydrolase, β-galactosidase, β-glucuronidase, N-acetyl-β-glucosaminidase, α-fucosidase, D-glucose, esculin ferric citrate, L-arabinose, D-mannose, N-acetyl-glucosamine, D-maltose, potassium gluconate, adipic acid, malic acid, phenylacetic acid
Negative tests with API	Acid phosphatase, α-galactosidase, α-glucosidase, β-glucosidase, α-mannosidase, potassium nitrate, L-tryptophane, L-arginine, urea, glatin(bovine origin), 4-nitrophenyl-βD-galactopyranoside, capric acid, trisodium citrate
Major cellular fatty acids	Iso-C_15:0_, antesio-C_15:0_, iso-C_17:0_, antesio-C_17:0_, iso-C_16:0_
Predominant menaquinones	MK-8 and MK-7
Major polar lipids	DPG, PE, PG, AL
Designation of the type strain	REN8^T^
Country of origin	China
Region of origin	Sichuan Province
Latitude	30°05′N
Longitude	102°54′E
Date of isolation	2019
Source of isolation	Pit mud
Strain collection numbers	JCM 33964^T^, GDMCC1.2213^T^
16S rRNA gene accession number	MW187781
Genome accession number	JADHDU000000000
DNA G+C content (mol%)	46.7

**Table 10:** Description of *Planococcus beijingensis* sp. nov.

Species name	*Planococcus beijingensis*
Genus name	*Planococcus*
Specific epithet	*beijingensis*
Species status	sp. nov.
Species etymology	bei.jing.en’sis. N.L. masc. Adj. beijingensis pertaining to Beijing, Chinese
Description of the new taxon and diagnostic traits	Cells are Gram-staining-positive, coccoid, aerobic and motile (1.0–2.0 μm in diameter, and occur singly, in pairs, in groups of three or in tetrads). Colonies on TSA are circular, slightly convex, glistening, smooth and light yellow in color after incubation for 48 h at 37°C. Cells are catalase positive and can hydrolyze starch and milk but not peptonisation. Growth occurs between 20°C and 45°C (optimal growth at 37°C) and at pH 5.0–12.0 (optimal growth at pH 6.0). Growth occurs with 0–15% NaCl (optimal growth with 1% NaCl).
Positive tests with Biolog	Utilize dextrin, D-maltose, D-trehalose, sucrose, D-turanose, α-D-lactose, N-acetyl-D-glucosamine, α-D-glucose, D-fructose, D-galactose, 1% Sodium lactate, D-serine, D-sorbitol, D-mannitol, D-arabitol, Glycerol, Troleandomycin, Glycyl-L-prolin, L-alanine, L-arginine, L-glutamic acid, L-serine, Lincomycin, Pectin, D-gluconic acid, Methyl pyruvate, D-lactic Acid methyl ester, L-lactic acid, Citric acid, Nalidixic acid, Lithium chloride, Potassium tellurite, α-hydroxy-butyric acid, Acetoacetic acid, Acetic acid, Formic acid, Aztreonam, Sodium butyrate. Weakly utilize D-mannose, 3-Methyl glucose, Inosine, D-glucose-6-PO4, D-fructose-6-PO4, D-aspartic acid, Gelatin, L-aspartic acid, L-histidine, L-pyroglutamic acid, D-galacturonic acid, L-galactonic acid lactone, D-glucuronic acid, Glucuronamide, Mucic acid, Quinic acid, D-saccharic acid, Tetrazolium violet, Tween 40, γ-amino-butryric, acid, Propionic acid, Sodium bromate
Negative tests with Biolog	D-cellobiose, Gentiobiose, Stachyose, D-raffinose, D-melibiose, β-methyl-D-glucoside, D-salicin, N-acetyl-β-Dmannosamine, N-acetyl-D-galactosamine, N-acetyl neuraminic acid, D-fucose, L-fucose, L-rhamnose, Fusidic acid, myo-inositol, Rifamycin SV, Minocycline, Guanidine HCl, Niaproof 4, Vancomycin, Tetrazolium blue, p-hydroxy-phenylacetic acid, α-keto-glutaric acid, D-malic acid, L-malic acid, Bromo-succinic acid, β-hydroxy-D,Lbutyric acid, α-keto-butyric acid
Positive tests with API	Esterase (C4), Esterase lipase (C8), naphthol-AS-BI-phosphohydrolase, urea, esculin ferric citrate, 4-nitrophenyl-β D-galactopyranoside, D-glucose, α-fucosidase, D-mannitol, D-maltose, potassium gluconate
Negative tests with API	Alkaline phosphatase, Lipase (C14), Leucine arylamidase, Valine arylamidase, Cystine arylamidase, Trypsin, α-chymotrypsin, Acid phosphatase, α-galactosidase, β-galactosidase, β-glucuronidase, N-acetyl-β-glucosaminidase, α-glucosidase, β-glucosidase, α-mannosidase, a-fucosidase, potassium nitrate, L-tryptophane, L-arginine, glatin(bovine origin), L-arabinose, D-mannose, N-acetyl-glucosamine, capric acid, adipic acid, malic acid, trisodium citrate, phenylacetic acid
Major cellular fatty acids	Iso-C_15:0_, antesio-C_15:0_, iso-C_17:0_, antesio-C_17:0_
Predominant menaquinones	MK-7, MK-8 and MK-6
Major polar lipids	DPG, PE, PG
Designation of the type strain	REN14^T^
Country of origin	China
Region of origin	Sichuan Province
Latitude	30°05′N
Longitude	102°54′E
Date of isolation	2019
Source of isolation	pit mud
Strain collection numbers	JCM 34410^T^, GDMCC 1.2209^T^
16S rRNA gene accession number	MW187783
Genome accession number	JADHDZ000000000
DNA G+C content (mol%)	45.1

## Data availability statement

The datasets presented in this study can be found in online repositories. The names of the repository/repositories and accession number(s) can be found in the article/Supplementary material.

## Author contributions

SH, JW, and LL: method. SH, and MH: data curation. SH: writing—original draft preparation. QR: writing—review and editing, supervision, and investigation. All authors have read and agreed to the published version of the manuscript.

## Funding

This work was supported by the National Key Research and Development Program of China (2018YFC1603606 and 2018YFC1603800).

## Conflict of interest

The authors declare that the research was conducted in the absence of any commercial or financial relationships that could be construed as a potential conflict of interest.

## Publisher’s note

All claims expressed in this article are solely those of the authors and do not necessarily represent those of their affiliated organizations, or those of the publisher, the editors and the reviewers. Any product that may be evaluated in this article, or claim that may be made by its manufacturer, is not guaranteed or endorsed by the publisher.

## Footnotes

^1^https://rast.nmpdr.org/rast.cgi
