# Supplementary material for: Two novel Planococcus species isolated from baijiu pit mud with potential application in brewing
Source: Front Microbiol. 2023 May 11;14:1139810. doi: 10.3389/fmicb.2023.1139810 (PMC10213732; doi:10.3389/fmicb.2023.1139810)
Supplement: Supplementary file 1 [file Data_Sheet_1.docx]

**Two novel *Planococcus species* isolated from *baijiu* pit mud with potential application in brewing**

^1^Key Laboratory of Brewing Molecular Engineering of China Light Industry, Beijing Technology and Business University, Beijing 100048, China.

**
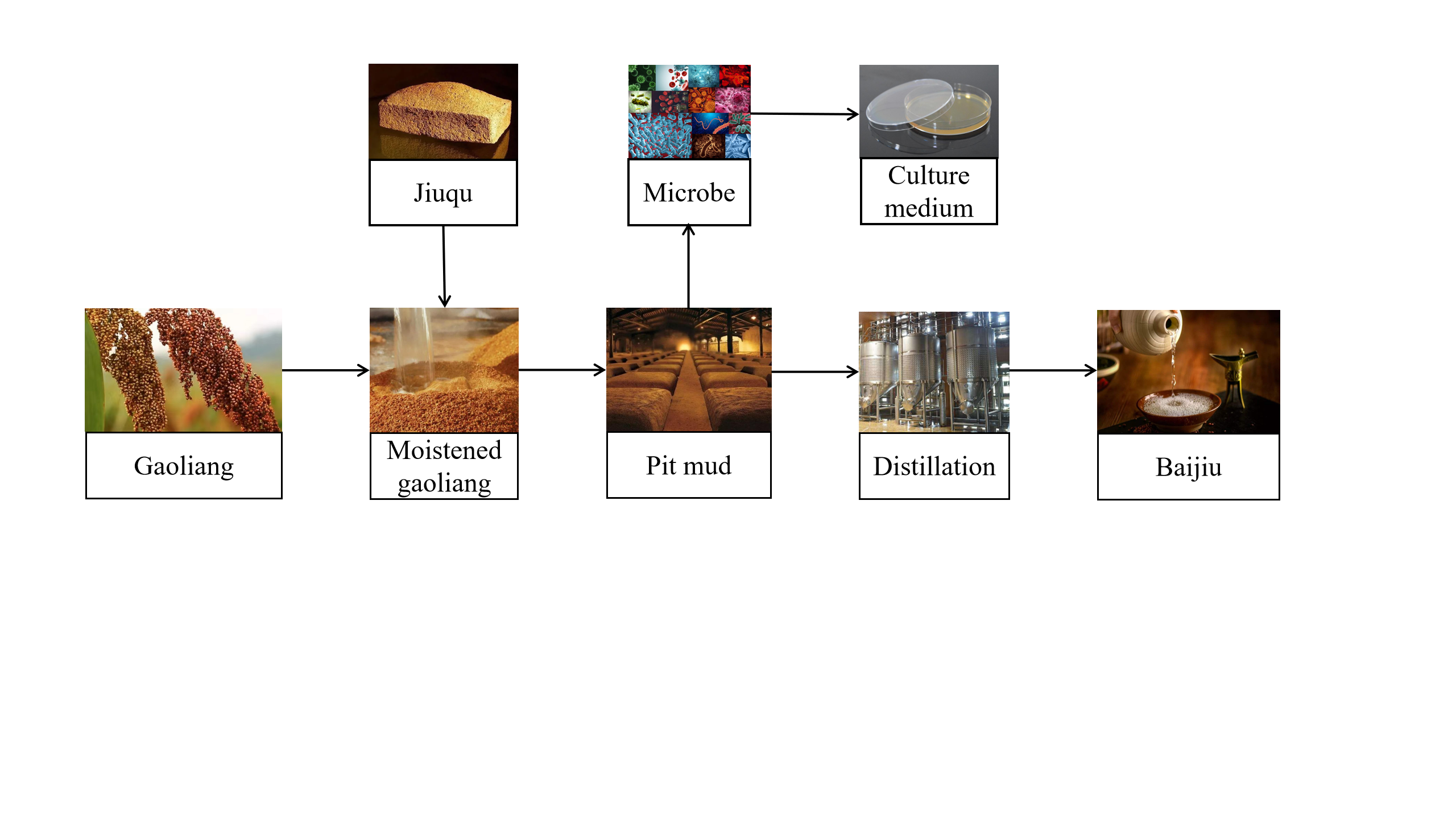
**

**Fig. S1** Summary of *baijiu* production process and isolation site of strains.

The gaoliang is crushed and added with appropriate amount of water, steamed, inoculated with jiuqu, fully fermented in the pit mud, and distilled to obtain *baijiu*. The pit mud contains rich microbial resources. The two new bacterial species in this study were isolated and cultured from the diluted pit mud.


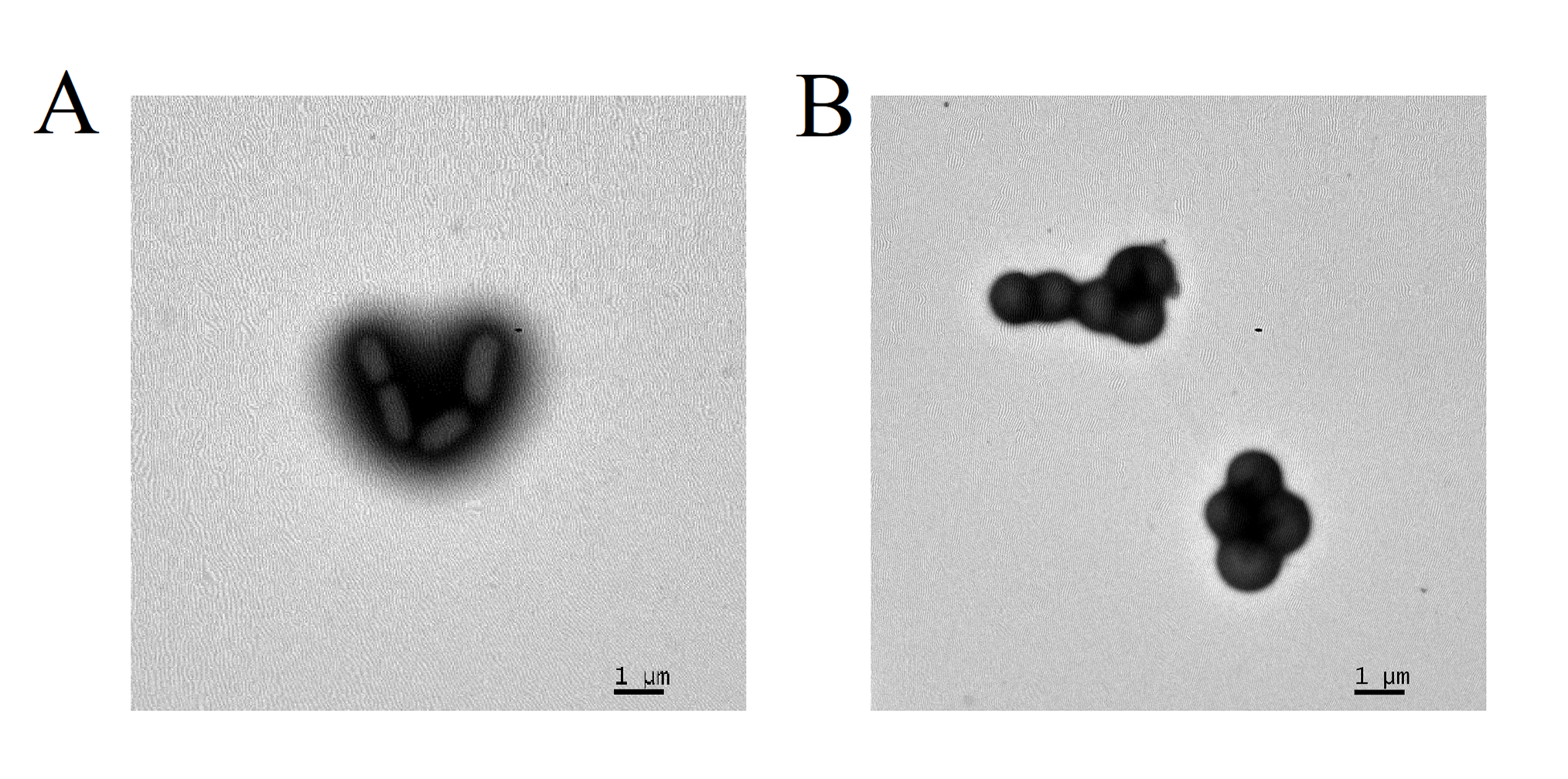
**Fig. S2** Transmission electron microscopy observation on REN8^T^ and REN14^T^. A: REN8^T^. B: REN14^T^.


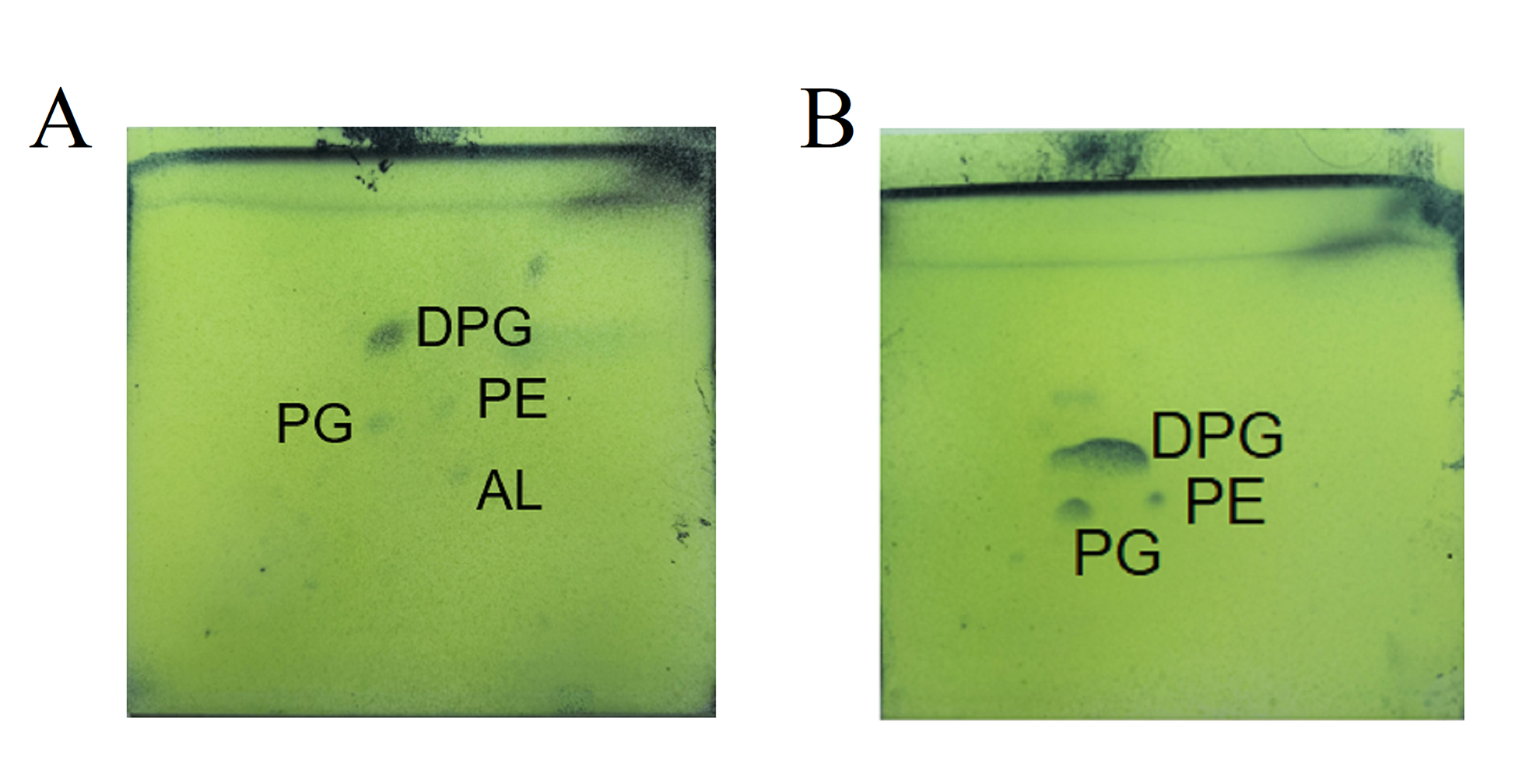
 **Fig. S3** Total polar lipids detection of strains REN8^T^ and REN14^T^. A: REN8^T^. B: REN14^T^.
